# Effects of cooperation between translating ribosome and RNA polymerase on termination efficiency of the Rho-independent terminator

**DOI:** 10.1093/nar/gkv1285

**Published:** 2015-11-23

**Authors:** Rui Li, Qing Zhang, Junbai Li, Hualin Shi

**Affiliations:** 1 State Key Laboratory of Theoretical Physics, Institute of Theoretical Physics, Chinese Academy of Sciences, Beijing 100190, China; 2 Institute of Chemistry, Chinese Academy of Sciences, Beijing 100190, China; 3 National Center for Nanoscience and Technology, Beijing 100190, China

## Abstract

An experimental system was designed to measure *in vivo* termination efficiency (TE) of the Rho-independent terminator and position–function relations were quantified for the terminator tR2 in *Escherichia coli*. The terminator function was almost completely repressed when tR2 was located several base pairs downstream from the gene, and TE gradually increased to maximum values with the increasing distance between the gene and terminator. This TE–distance relation reflected a stochastic coupling of the ribosome and RNA polymerase (RNAP). Terminators located in the first 100 bp of the coding region can function efficiently. However, functional repression was observed when the terminator was located in the latter part of the coding region, and the degree of repression was determined by transcriptional and translational dynamics. These results may help to elucidate mechanisms of Rho-independent termination and reveal genomic locations of terminators and functions of the sequence that precedes terminators. These observations may have important applications in synthetic biology.

## INTRODUCTION

Two mechanisms of transcriptional termination, including Rho-dependent and -independent termination (or intrinsic termination), have been described in bacteria (1,2). Rho-dependent termination relies on destabilization of transcription complexes by the regulatory protein factor Rho, which binds at the *rut* (Rho utilization) site in nascent mRNA, moves in the 5′–3′ direction along mRNA as a molecular motor, and terminates transcription upon contact with RNA polymerase (RNAP) (3). Rho-independent terminators consist a short DNA sequence that encodes a motif in the nascent transcript with standard secondary structures: a GC-rich hairpin followed by a U-tract (1,2). Rho-independent terminators can terminate transcription without the assistance of auxiliary factors. Weaker base pairing between the mRNA U-tract and the template DNA A-tract lowers the stability of the transcription complex and slower transcriptional elongation at U-tract allows time for GC rich hairpin folding, which destabilizes the transcription complex (1). Although the details of transcription complex destabilization remain elusive, three mechanisms have been proposed. In the ‘forward translocation model’ (4,5), it has been suggested that hairpin folding causes RNAP forward translocation and shortens the RNA–DNA hybrid, causing destabilization of the transcription complex. In contrast, the ‘hybrid-shearing model’ (5,6) suggests that the RNA hairpin acts by extracting RNA from the transcription complex, and ‘the allosteric model’ (7) involves invasion of RNAP by the RNA hairpin, leading to extensive conformational changes of the transcription complex without forward translocation. Hairpin folding is a key step in the termination process, and single molecular experiments showed that inhibition of hairpin folding enables RNAP to read through the terminator (5).

Although bacterial genomes are compact, most terminators are not directly located downstream of stop codons of upstream genes, and lengths of sequences between stop codons and terminators vary, ranging from zero to several hundred base pairs. Most stop codon–terminator distances are <50 bp, and some terminators overlap with upstream genes (8–10). Based on sequential and structural conservation of Rho-independent terminators, bioinformatic analyses have revealed numerous putative terminators in coding regions (8,9). However, if these terminators are functional, non-stop RNA would be produced. Hence, further studies are required for determining terminator positions and factors affecting efficiency.

Sequence contexts also influence terminator functions, and terminators followed by GC-rich sequences have higher termination efficiencies (TEs) than those followed by AT-rich sequences (7). However, effects of upstream sequences remain poorly understood, and numerous terminators have A-tracts preceding the hairpin (8) and effects of additional base pairing of A-tracts with U-tracts remain controversial (11–13). Terminators with A-tract preceding the hairpin can function as bidirectional terminators, i.e. they can terminate transcription in both strands (8,14,15). However, even with a symmetrical structure, some bidirectional terminators have non-equal TEs in different directions (16). Thus, multiple factors, such as terminator positions and upstream and downstream sequences, may affect TEs in conjunction with terminator sequences.

In bacteria, transcription and translation occur simultaneously in the same compartment, and ribosomes initiate translation of nascent RNA concurrently with transcription. Thus, the transcription–translation coupling is a specific feature of prokaryotic gene expression and plays essential roles in many aspects of regulation. During transcriptional attenuation, this coupling is precisely regulated by transcriptional or translational pausing, which switches terminators on or off (17,18). The translating ribosomes can repress terminator function by directly inhibiting terminator hairpin folding (16,18) or by modifying mRNA secondary structure into the competitive anti-termination form (19). The first translating ribosomes on newly synthesized RNA can inhibit transcriptional backtracking during transcriptional elongation (20,21). Hence, transcription–translation coupling might influence the efficiency of general terminators.

Numerous terminators are located in intergenic regions of operons (10,22,23), and these terminators coordinate expression levels of different genes within the operon (24), necessitating a strict control of termination efficiency. Widespread antisense RNAs (25) have multiple regulatory functions (26) and among other mechanisms, readthrough of terminators generates antisense RNAs (27). These processes may also be affected by the transcription–translation coupling.

In the present study, we investigated the relation between terminator positions and their functions using an *in vivo* experimental system for determining the termination efficiency of the Rho-independent terminator. The well-studied lambda phage terminator tR2 (5) was inserted between two distinct fluorescence protein genes, and termination efficiency was quantified by measuring the relative fluorescence. Insertion of various lengths of sequence between the stop codon of the upstream gene and terminator produced a series of constructs with tR2 located at different distances from the stop codon. Other constructs contained terminators in the coding regions, and subsequent comparisons of TEs revealed that relations between positions and terminator functions are sequence specific. Finally, we propose a model of stochastic coupling between ribosomes and RNA polymerases that explains this relation.

## MATERIALS AND METHODS

### Experiment system construction

**Figure 1.**
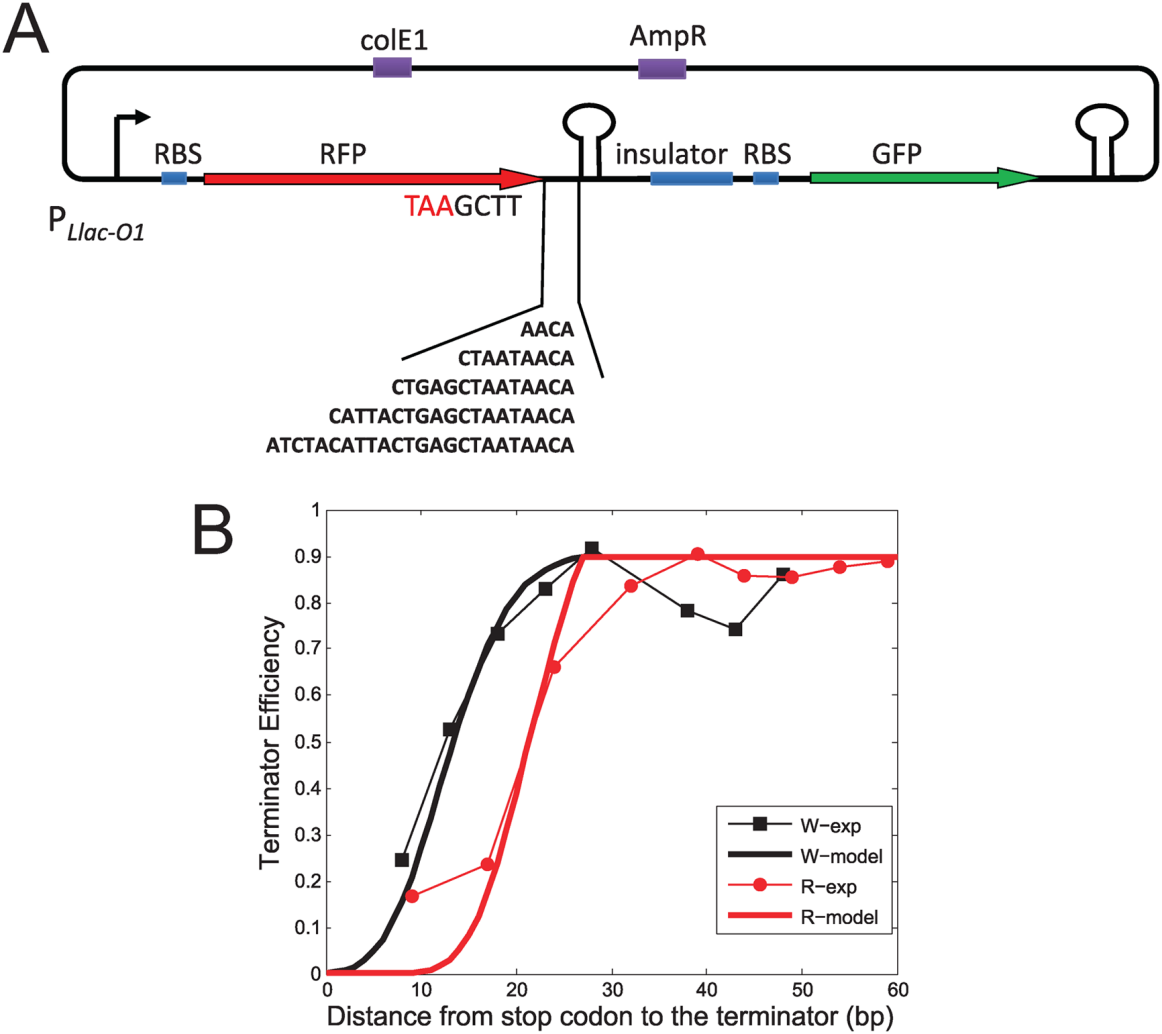
Experiment system and measured termination efficiency of the tR2 terminator located downstream of the RFP gene. (**A**) Operon design for measurements of termination efficiencies. The terminator is located between RFP and GFP, the ‘TAA’ stop codon of RFP is indicated in red, and the insulator sequence was inserted to prevent the effects of upstream sequences on translation of GFP. Sequences of various lengths were inserted between upstream RFP and terminator. (**B**) TE–distance relations of W-series (black square, experiment results; black thick line, model fitting result) and R-series (red dot, experiment data; red thick line, model fitting result); black and red thin lines are presented as visual guides; distance is indicated by the length of the spacer between the last base pair of the stop codon and the first nt of the terminator hairpin stem. Experimental data are listed in Supplementary Table S4. Model fitting see Equation (3).

Initially, we constructed an operon with the plasmid pZE12 using two different fluorescence protein genes *RFP* and *GFP* (Figure 1A), driven by promoter *P_Llac-O1_* which can be induced by isopropyl β-D-1-thiogalactoside (IPTG) (28) and placed the terminator tR2 in the intergenic region between RFP and GFP genes. We also constructed plasmids with the terminator in the coding region. To avoid changing the ribosome biding site (RBS) strength of GFP in the presence of various terminators and upstream sequences, we inserted a 79-bp random sequence between the terminator and the RBS of GFP as an insulator (Figure 1A).

### Medium, growth and measurements

Plasmids were transformed into the *Escherichia coli* strain BW-RI (29), which constitutively expresses the regulator *LacI*. Cells with plasmids were cultured to mid-log phase in M9 media at 37°C in the presence of 0.5% glucose and appropriate antibiotics. Cultures were diluted (1:250) into fresh media and cultivated overnight, and were then diluted to OD_600_ = 0.002 in fresh media containing antibiotics, glucose, and 10-mM IPTG at saturation concentration for P*_Llac-O1_* promoter (28) in wells of costar 48-well plate shaking at 170 rpm. Cell growth was measured by OD_600_ and fluorescence intensities were measured using a Wallac Victor3 1420 multilabel counter (PerkinElmer Life Sciences) every 30 min during exponential growth. Measurements were repeated at least three times. Prior to real-time polymerase chain reaction (PCR) experiments, total RNA was extracted using TransGen EasyPure RNA Kits and residual DNA was removed using Ambion Turbo DNA-free DNase. Subsequently, 1 μg total RNA was used for cDNA synthesis using TaKaRa PrimeScript II 1st Strand cDNA Synthesis Kits, and cDNAs from 12-ng RNA (for RFP and GFP) or 0.12-ng RNA (for rrsB) were used for quantification in real-time PCR with Roche FastStart Universal SYBR Green Master mix. Real-time PCR was performed using an Eppendorf mastercycler ep gradient realplex^4^ real-time PCR system. (For details see SI ‘Materials and Methods’ section.)

### Calculation of termination efficiency

Protein concentrations were used to determine mRNA levels in our study. After measuring fluorescence intensities, TEs of terminators in each plasmid were calculated using the following equation:

(1) \begin{equation*} {\rm TE}_{tR2} = 1 - \frac{{P_{{\rm GFP}_{tR2} } /P_{{\rm GFP}_{{\rm reference}} } }}{{P_{{\rm RFP}_{tR2} } /P_{{\rm RFP}_{{\rm reference}} } }}, \end{equation*}

where $P = \mu f\left( {1 + \frac{{\mu \tau }}{{ln2}}} \right)$ is the synthesis rate of fluorescence protein per cell (30,31), *μ* is the cell growth rate,$f = \frac{{\partial F}}{{\partial OD}}$, *F* is the measured fluorescence intensity and *τ* is the maturation half-time of fluorescent proteins. The reference is the plasmid I21 without a terminator between RFP and GFP genes, and we set TE*_I_*_21_ = 0. We use RFP expression as the indicator of transcription strength of the promoter. Due to the fact that translation efficiency and fluorescence brightness may be different for two proteins, we use RFP and GFP synthesis rate ($P_{{\rm RFP}_{{\rm reference}} }$ and $P_{{\rm GFP}_{{\rm reference}} }$) of cell with plasmid I21 as normalization respectively. Then, $P_{{\rm GFP}_{tR2} } /P_{{\rm GFP}_{{\rm reference}} }$ and $P_{{\rm RFP}_{tR2} } /P_{{\rm RFP}_{{\rm reference}} }$ represent relative mRNA levels of fluorescence genes to the reference after tR2 terminator was inserted. TEs of plasmids with modified RFP genes were calculated using the following equation:

(2) \begin{equation*} {\rm TE}_{{\rm GFP}_{tR2} } = 1 - \frac{{P_{{\rm GFP}_{tR2} } }}{{P_{{\rm GFP}_{{\rm reference}} } }}. \end{equation*}

If the stability of mRNA changed because of the modifications, results got from Equation (2) would not be accurate. In our study, for those plasmids with modified RFP gene, quantitative real-time PCR experiments indicate that expression levels of RFP mRNAs are almost not changed (Supplementary Table S8). This means TEs calculated from Equation (2) are usable for those plasmids. For those plasmids with intact RFP gene, RFP expressions are relatively the same (see Supplementary Figure S1A) with the reference and Equation (2) may be a good approximation for Equation (1). Some measured TEs were confirmed at the mRNA level using quantitative real-time PCR (Supplementary Table S8).

## RESULTS

### Gradual tuning of TEs by position

We first studied terminators located in the intergenic region and investigated the influence of the distance between the terminator and the stop codon of upstream genes on functions of terminators. From a sequence chosen for insertion, we progressively shortened the 5′ end to generate a series of insertion sequences (see Supplementary data ‘Supplementary Materials and Methods’ section). Then these sequences of different lengths were inserted between RFP stop codons and tR2 terminators (Supplementary Table S3). Besides the length, the sequence detail may also influence termination efficiency. To study this sequence specificity, we constructed R- and W-series plasmids by inserting two different series of sequences between RFP gene and tR2 terminator. For W-series plasmids, insertion sequences (Supplementary Table S3) were difference lengths of natural sequences preceding tR2 terminators in the lambda phage genome, with the exceptions of W48-up1 and W48-up2 in which the translation initiation site introduced in W48 was silenced by mutating the RBS and start codon, respectively. The effects of this translation initiation site in W48 will be shown and discussed later. For R-series, the insertions (Supplementary Table S3) comprised various lengths of a sequence, which was chosen from computer-generated random sequences. The criteria are that the insertion itself has relatively less secondary structures and cannot form structures that inhibit terminator hairpin folding when linked to the terminator. The distances between stop codons and terminators in W- and R-series constructs ranged from 8 to 48 bp and 9 to 59 bp, respectively.

All strains carrying plasmids of W- and R-series constructs had comparable RFP expression levels to the reference strain (as in Supplementary Figure S1B). TEs were calculated for each plasmid and displayed in Figure 1B, several TEs were confirmed at the RNA level using quantitative real-time PCR (Supplementary Table S8). The terminator tR2 was almost fully repressed when distances to the stop codons were short, but gradually increased to the maximal value of ∼0.9 with increasing distances. In constructs with distances beyond 30 bp, TEs fluctuated around the maximum. However, despite the tendency to increase with larger distances, TEs differed for W- and R-series constructs, suggesting that the TE–distance relation is sequence specific. For example, W18 had a distance of 18 bp and a TE of ∼0.8, whereas R17 had a distance of 17 bp and a TE of only ∼0.12. However, sequence specificity was diminished with distances of more than 30 bp, and plasmids of both series had similar maximal TEs of ∼0.9 (Figure 1B). The common tendencies and differences between these two series can be explained by coupling of transcription and translation (details will be discussed below). Roland *et al*. (18) also found similar effects when pryBI attenuator are studied, however TE–distance curve they measured is very steep, 3 bp increasing of the distance at the repression boundary almost relives 60% of repression.

### Ribosomes repress terminators near stop codons

**Figure 2.**
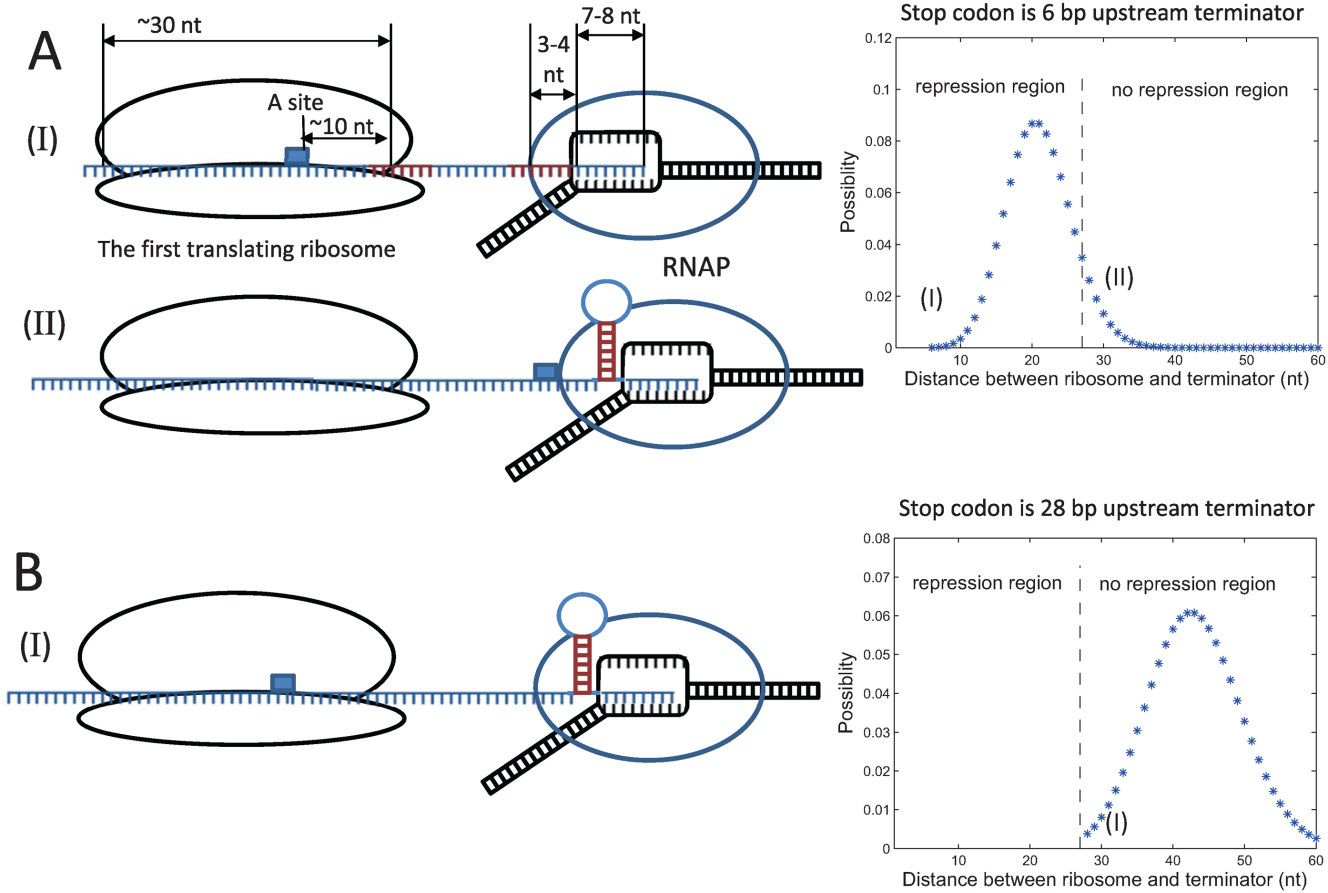
Cooperation between ribosomes and RNAP determines TE of terminators. DNA is shown in black, mRNA is shown in blue, the small blue rectangle indicates the stop codon of RFP, the stem of terminator hairpin is in red. (**A**) Two snapshots of coupling between transcription and translation when terminator is 6 bp downstream of stop codon. Subplots (I) and (II) represent ribosome at different positions can or cannot repress terminator hairpin folding. (**B**) The snapshot shows even the closest ribosome at stop codon cannot repress terminator when terminator is far enough to stop codon. The assumed ribosome distributions are plotted in the right respectively, using the parameter of R-series, details see description of Equation (3), positions of snapshots in the distribution are labeled in the plots.

Considering translation and transcription are coupled together in bacteria, detailed analysis of configurations of translational and transcriptional complexes suggest that first translating ribosome may repress terminator function. Ribosomes profiling experiments (32) showed that the ribosome spans ∼30 nt of mRNA, and protects ∼10 nt of RNA downstream of the translation active site (A site) (33). RNAP protects ∼4–5 nt of mRNA in addition to the 7–8 nt base paired with template DNA (19,34,35). In the ‘allosteric model’ (7) of terminators, upon arrival of RNA polymerase at the poly-U termination site, successful termination requires folding of the palindrome mRNA sequence into a full hairpin inside RNAP. In contrast, invasion of RNAP by the hairpin is not necessary in ‘forward-translocation model’ (4,5) and ‘hybrid-shearing model’ (5,6). Closure of the final 2–3 bp of the hairpin is reportedly essential for termination (5,7,19,36), suggesting that the presence of at least 10–15 nt of free mRNA between the ribosome A site and the first base of the hairpin allows full folding of the terminator hairpin. Experimental results also show that ribosome and RNAP can be linked by a NusE:NusG complex (37,38) or other transcription factors (39) after transcriptional initiation (40). Thus, considering the steric effects of these links (37,39), the minimal distance between ribosome and terminator required for full folding of the terminator hairpin may be larger. Moreover, the position of the stop codon determines the possible closest distance between RNAP at the terminator site and ribosomes. Sufficient distances between the terminator and the stop codon of the upstream gene can keep sufficient distances between tailing ribosomes and RNAP upon arrival at the termination site (Figure 2B). Otherwise, as shown in (I) of Figure 2A ribosomes may sequester a part of mRNA, which would repress termination hairpin folding, allowing RNAP to read through the terminator. Figure 1B indicates repression of terminators that are located several base pairs downstream of stop codons of upstream genes.

**Figure 3.**
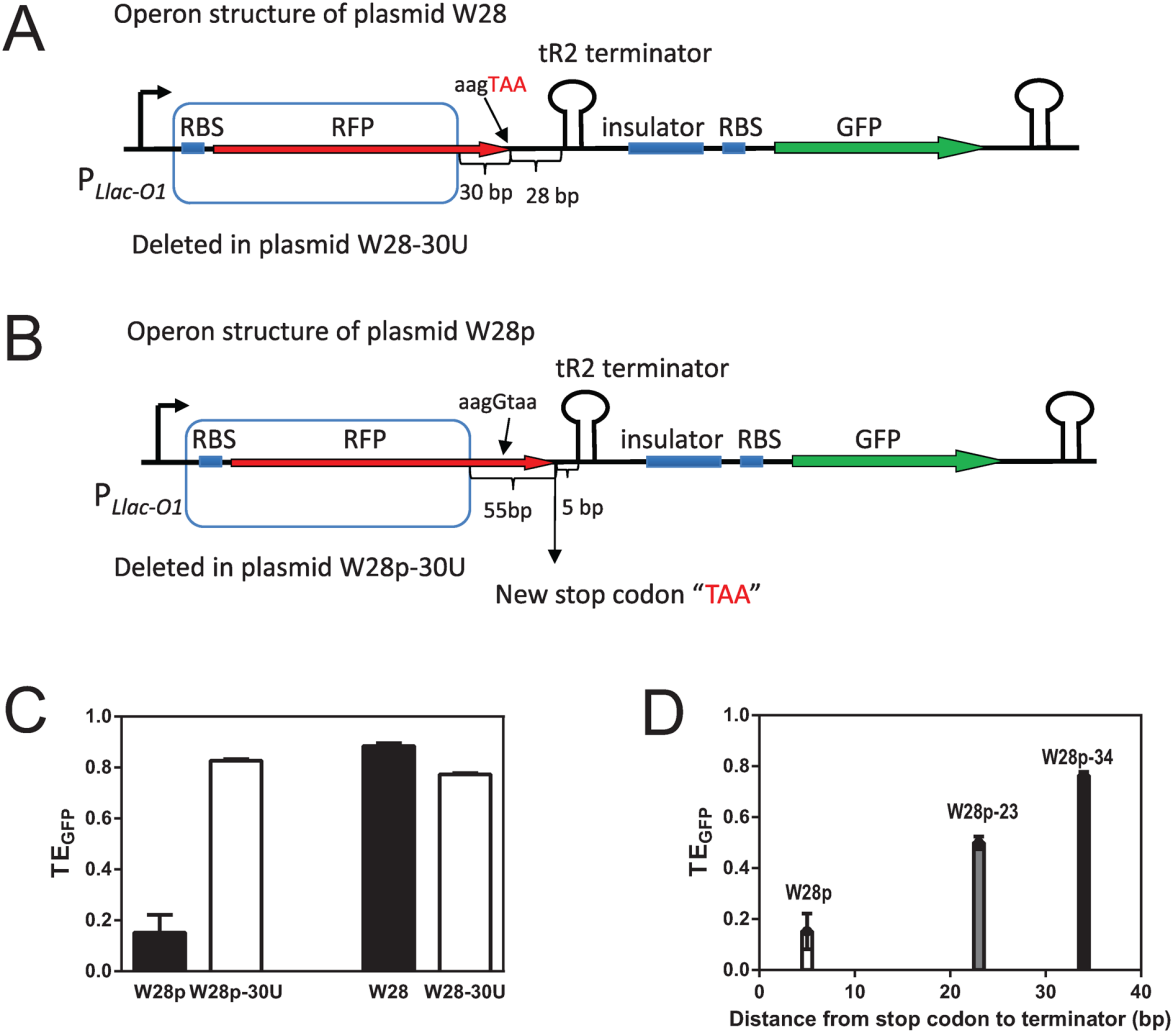
Effects of uncoupling translation of RFP from transcription on TE. (**A** and **B**) Operon structure of plasmid W28 and W28p. After inserting one ‘G’ base pair upstream of the stop codon ‘TAA’ of the RFP gene in the plasmid W28, the new stop codon was located 5-bp upstream of the terminator in W28p, stop codons are labeled in red. The plasmid suffix ‘-30U’ indicates uncoupling of translation of RFP by deletion of the RBS and the upstream part of the coding sequence of RFP. Deleted sequences for plasmids W28–30U and W28p-30U boxed in (A) and (B) are same; the last 30 bp sequence of original RFP gene were intact. (**C**) TE of terminators when translation are uncoupled. (**D**) Inserting sequence between tR2 terminator and stop codon of modified RFP gene in W28p relieves repression of terminator. Numerical data are listed in Supplementary Tables S4, S6 and S7.

Data from the present and previous study (18) suggest that differences of TE of tR2 terminators at different positions reflect differing degrees of ribosome repression. To test this hypothesis in our system, changes of TE were determined upon removal of RFP translation by deleting the RBS of RFP and its upstream coding region to avoid Rho factor function, and the last 30 bp was preserved to maintain the local sequence preceding the terminator. Due to the fact that different insertion sequences may lead to different secondary structures, we didn't directly do this deletion for the W-(or R-)series plasmids. Instead, we first constructed a new plasmid, W28p, which has the minimal sequence difference with W28, but with a very short distance between the terminator and stop codon. By inserting only one additional ‘G’ upstream original stop codon ‘TAA’ in plasmid W28, we move the stop codon from 28 to 5-bp upstream of the terminator in plasmid W28p (Figure 3B). Compared with WT *RFP* genes, modified *RFP* in plasmid W28p has a different 3′-end and different fluorescence properties. Thus, TE_GFP_ (Equation (2)) was used to measure terminator efficiency, which was reduced from ∼90% to <10% after insertion of this ‘G’ (Figure 3C).

In comparisons of W28 and W28p (Figure 3C), ribosomes directly repressed terminator function when the stop codon was only a few base pairs upstream of the terminator. Moreover, subsequent extending inserted sequences between stop codon and terminator in W28p led to increasing TE (Figure 3D). Thus, in further experiments, we uncoupled translation and transcription by deleting the RBS and the upstream coding sequence of *RFP* gene but kept the last 30-bp coding sequence of *RFP* (plasmids denoted with the suffix -30U, see Figure 3A and B). In Figure 3C, we demonstrated that terminator repression was completely abolished by uncoupling of translation from transcription.

### Stochastic coupling of ribosome and RNAP

Compared with very steep TE–distance relation with a sharp boundary of repression for the terminator inside of pryBI leader peptide (18), the smooth TE–distance relations we measured indicate the dynamics of ribosome and RNAP are different in our system. As discussed above, terminator function requires sufficient distance between the first translating ribosome and RNAP. Thus, for terminators located close to the stop codon of the upstream gene, suppression reflects the distance between RNAP and the first translating ribosome. Proximal ribosomes prevent proper folding of the terminator hairpin, allowing readthrough by RNAP (I of Figure 2A). In contrast, distal ribosomes allow proper terminator function (II of Figure 2A). Hence, because the elongation processes of both ribosomes and RNAP are stochastic (41–44), the distance between RNAP and the first translating ribosome upon arrival of RNAP at the terminator site is random, and the distribution of ribosome determines the shape of TE–distance relation. Ribosome stops at the stop codon, thus the lower limit of this distance is determined by that between the terminator and the stop codon. When this stop codon–terminator distance is sufficient (>27 nt in the present system), the ribosome at the stop codon with the shortest distance to RNAP cannot repress terminator hairpin folding, leading to maximal TE (Figure 2B). Hence, the statistical distribution of ribosome positions upon arrival of RNAP at the termination site leads to gradual increases in termination efficiency with increasing distances between stop codons and terminators, and is maximal when the distance is larger than the threshold (Figure 1B). The distribution is determined by dynamics of ribosome and RNAP together, and both dynamics are sequence specific (41–44). As a result, different insertions in W- and R-series give different TE–distance relation (Figure 1B). In pryBI attenuator, transcription and translation paces are strictly controlled by transcriptional pausing sites (18), this is the reason that the measured TE–distance curve is quite steep. Experiments of Larson *et al*. (20) indicate that this distribution of ribosome positions is independent of translation strengths of upstream genes if the RBS is not too weak. In our experiments, changes in the RBS of RFP resulted in halved translation of RFP with little changes in TE (Supplementary Figure S2). Thus, the relation between TE and ribosome distribution can be described as follows:

(3) \begin{equation*} {\rm TE}(d) = {\rm TE}_0 [1 - {\rm CDF}(\alpha ,\lambda (d))], \end{equation*}

where *d* is the distance from the stop codon to the first base of the terminator hairpin and *CDF*(*α, λ*(*d*)) is the cumulative distribution of the first translating ribosome upon arrival of RNAP at the terminator site, i.e. the possibility that the distance between the first translating ribosome and the first base of the terminator hairpin is less than *α* nt, and *λ* indicates parameters of the distribution, which are dependent on *d*. In our model, details of transcriptional and translational termination processes are disregarded, we only suppose ribosomes cannot repress terminator function if the distance to the first base of terminator hairpin is larger than *α* upon arrival of RNAP at the terminator site (in our system *α* = 27 nt), and TE = TE_0_ (TE_0_ is the maximal TE, for tR2 terminator, we useTE_0_ = 0.9), otherwise the ribosome would hinder hairpin folding resulting in repression of the terminator. Thus, in our system a Poisson distribution with parameter *λ* was used to fit the ribosome distribution and the position of stop codon determined the lower bound of this distribution. Accordingly, *λ* = *d* + 15 (nt) was used for W-series plasmids, and *λ* = *d* + 7 (nt) was used for R-series plasmids in our model, the different values of *λ* originate from sequence specificity of stochastic coupling of transcription and translation. This model fits well with the experiment results as shown in Figure 1B.

### Terminators in coding regions

Based on sequence and structure conservation, bioinformatic methods predicted numerous putative terminators located inside the open reading frame (ORF) of protein-coding genes (8). Termination of transcription at these putative terminators should be suppressed to prevent the production of non-stop mRNAs. Moreover, in the absence of transcription–translation coupling, the translocation of ribosomes along mRNA would be much faster than that of RNAP along DNA (20). Hence, the first translating ribosome needs to wait behind RNAP most of the time, leading to similar average elongation speeds of RNAP and the first ribosome. Closely-following ribosomes have also been shown to repress transcription pausing (20). Recent ribosome profiling experiments showed non-unified translational elongation speeds, and that translation pausing sites is dominant by anti-Shine–Dalgarno sequences in coding regions (41). Severe translation pausing would temporarily increase the distance between RNAP and the first translating ribosome, and would prevent terminator repression by the ribosome.

**Figure 4.**
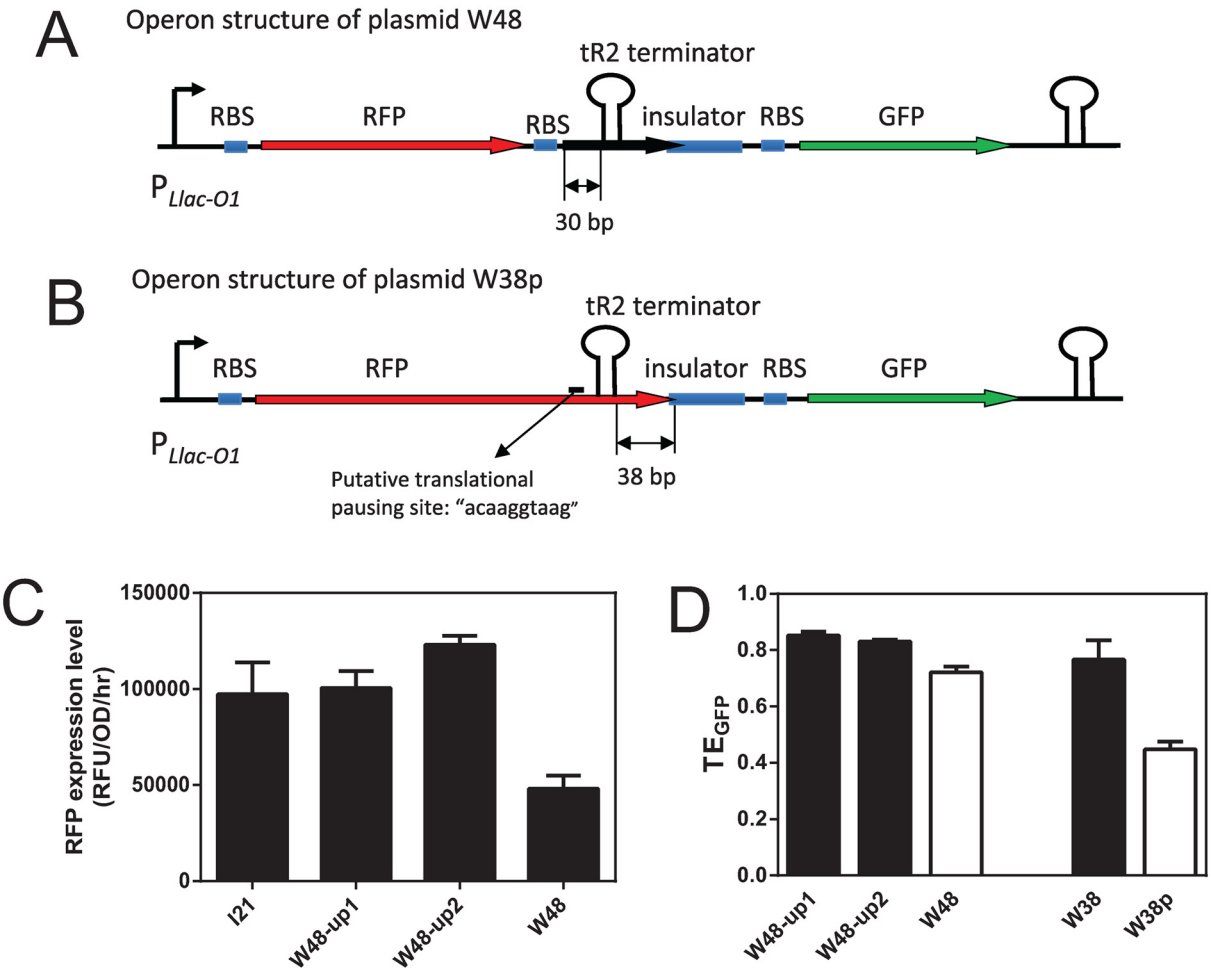
Terminators in coding regions. (**A**) Operon structure of the plasmid W48. (**B**) Operon structure of the plasmid W38p. After inserting one ‘G’ bp upstream of the stop codon of the RFP gene in plasmid W38, the new stop codon was located 38-bp downstream of the terminator. The putative translational pausing site was predicted using algorithm from Li *et al*. (41). (**C**) RFP expression level and W48 and -up1 and -up2. (**D**) TE of terminators in coding regions; In plasmids W48-up1 and W48-up2, RBS site or ‘AUG’ were mutated to silence the translation initiation site (sequence see Supplementary Table S3). Numerical data are listed in Supplementary Tables S4 and S6.

We first constructed a terminator inside of the first 100 bp of coding region and study its TE. Because a translation initiation site of *NinA* protein (45,46) is present in the insertion sequence of W48 plasmids, we introduced an ORF into plasmid W48 to create a tricistronic operon (Figure 4A) and the terminator tR2 is located only 30-bp downstream of the start codon ‘AUG’ and 16-bp upstream of the stop codon of the second ORF. Thus for mRNAs that are terminated at the tR2 terminator in W48, ribosomes from this translation start site would not meet any stop codon. This non-stop translation complex will trigger trans-translation systems to introduce ribosome-rescue and active degradation of non-stop mRNA, thus lowering the stability of mRNA (47). This is consistent with present measurements which showed that RFP expression from W48 was approximately half that of the reference plasmid I21 (Figure 4C). Hence, because clear decrease in RFP expression indicates that this translation initiation site is not weak, we silenced the translation start site to see the effect on TE by mutating the RBS or start codon of the second ORF in plasmid W48 (mutation sites see Supplementary Table S3), the resulting plasmids were W48-up1 and -up2. RFP expression levels recovered to similar levels as that from the reference plasmid I21 (Figure 4C). Moreover, comparisons of W48 and W48-up1 (or -up2) showed that the TE of the terminator in W48 was only lowered by ∼10% by translation repression, and the terminator retained most of its efficiency (Figure 4D and Supplementary Table S6). The average time needed to initiate translation for *E. coli* cells at various growth rates is between 1 and 2 s (48,49). Larson *et al*. (20) suggested that RNAP would be 20–40 nt away (20,32) from the RBS during this time window of translation initiation, and that the first translating ribosome would catch up with RNAP in ∼100–200 nt of newly synthesized mRNA, and then closely follows RNAP to the stop codon. Moreover, codons are comparatively rare at the 5′-end of the coding sequence compared with the downstream sequence, and ribosome elongation is relatively slow in this region (32). Thus, the ribosome is unlikely to catch up with RNAP in the first 100 bp (15,20), allowing the terminator in the first 100 bp of coding regions to function without ribosomal repression. Neverthless specific mechanisms of coupling of translation with transcription exist, for example, transcription pausing sites can couple translation with transcription in the short leader peptides of the pyrBI operon (18) and several other amino acid synthesis operons (50,51).

To investigate terminators in later part of the coding sequence, we extended coding region of the RFP gene by adding a ‘G’ before the stop codon ‘TAA’ in the plasmid W38, and the resulting stop codon was located 38-bp downstream of the tR2 terminator U-tract (Figure 4B). As shown in Figure 4C, TE_GFP_ for W38p was not total abolished and was decreased to 40% of W38, suggesting that translation pause sites partially uncouple transcription and translation, and decrease the chances of ribosomal suppression to approximately half. *In vitro* experiments of Wright *et al*. (16) showed that the function of a terminator in the downstream part of the coding region was completely abolished, which indicates a close coupling between translation and transcription. In agreement with our model, TE was partially restored in their system by slowed translation elongation in the presence of the antibiotic fusidic acid (16). These observations indicate that dynamic details of ribosomes and RNAP may determine the function of terminators located downstream of the first 100 bp of the coding sequence.

## DISCUSSION

Predicting the TE of a terminator is a challenge for quantitative biology. There are many factors influencing TE of Rho-independent terminator, which include the length and composition of U-tracts (11,52–54), compositions of hairpin stems (5,52) and especially sequences of closing basepairs of the hairpin (5,36). Investigations into functions of preceding sequences have been focused on the ∼10-nt A-tract (12,53). Terminators with A-tracts can function as bidirectional terminators, and the A-tract functions as a T-tract on the other strand. However, it remains unknown whether the extended basepairing introduced by A-tracts can increase termination (12,53). The influence of transcription–translation coupling on Rho-independent terminator efficiencies is predominantly appreciated in attenuator mechanisms (17,18).

In the present study, we investigated Rho-independent terminator functions at various positions, and focused on those located downstream of the 3′ ends of genes, which predominate in bacterial genomes. TEs were measured for the well-characterized lambda phage terminator tR2 with a dual-fluorescence protein expression system. We also constructed terminators inside of ORFs. We elucidated the relation between terminator position and terminator efficiency. This relation reflected stochastic coupling between ribosomes and RNAP. Fine tuning of TE could be exploited in synthetic biology applications, and the present techniques offer a simple method for manipulation of TEs without changing terminator sequences.

### TEs of terminators at different positions

Thousands of putative terminators have been located inside coding regions in *E. coli* by bioimformatic methods (8). In addition to those with regulatory functions, transcriptional termination at these terminators may produce non-stop mRNAs, thus wasting cell resources and burdening cell trans-translation systems (47). The present data show that terminators in the first 100 bp of coding regions are much less likely to be suppressed than those in downstream regions because translation still is not coupled with transcription yet. Accordingly, the terminator of the *pyrBI* attenuator is located within the 135-bp long leader peptide gene, and is not repressed in the presence of normal UTP concentrations (18). For several amino acid synthesis operons, leader peptide genes are around 100 bp (see Supplementary Table S9), transcription pausing sites in the leader peptide gene ensure the coupling and then attenuation can be regulated (50,51).

Repression of terminators in downstream coding regions by closely-following ribosomes may prevent production of non-stop mRNAs by putative terminators, and evolutionary selection of upstream sequences of the putative terminator may have ensured appropriate repression by proximal RNAP and ribosomes. Rho-independent terminators in coding regions may also function in the presence of frameshift mutations that cause premature translation termination. Hence Rho-independent terminators in coding region may play important roles in translational surveillance, which is similar to that Rho factor terminate the transcription of mRNA which is not translated properly (2).

Data from experiments with W- and R-series plasmids showed severe ribosomal repression of terminators located downstream of RFP ORF when the distance between the stop codon and the terminator was short. However, TEs gradually increased with this distance (Figure 1B). In our system, continuous increases in TE without abrupt alterations of TE–distance relations (Figure 1B) may originate from stochastic movements and then stochastic coupling of ribosome and RNAP (Equation (3) and Figure 2). Stochastic coupling of transcription and translation during elongations also have been surveyed theoretical by Mäkelä *et al*. to understand its effects on gene expression and noise (55).

In accordance with our stochastic model, different transcriptional or/and translational elongation dynamics will cause different coupling, then different TE. In the study of *pryBI* attenuator, Roland *et al*. (18) found a very steep TE–distance relation, which was corresponding to strictly coupling of ribosome and RNAP regulated by transcription pausing sites. This is the biggest different between our system and pryBI attenuation system (18). According to our model, decreases in translation rates will increase the distance between ribosome and RNAP upon arrival of RNAP at the termination site, and lead to higher TEs for terminators located near stop codons. Wright *et al*. (16) showed *in vitro* that decreased translation elongation rates in the presence of the antibiotic fusidic acid led to increases in TE of a terminator with 7 bp distance to the upstream gene. Moreover, *in vitro* experiments in which translation was not coupled with transcription showed that increases in transcription elongation rates decreased TE (56,57). However, when translation and transcription are coupled together, the stochastic coupling model above suggests that increasing transcription rates improves TE. Hence, the effects of transcription rates on TEs of terminators will be determined by the combination of these influences. In addition, increases in promoter activity can accelerate transcription elongation (56), and Chen *et al*. (52) showed that TEs of several terminators indeed increased with activities of upstream promoters.

Our results can help to understand why terminators are all have a relative >20 bp distances (Supplementary Table S9) to upstream leader peptide genes in amino acids synthesis attenuators. Transcription termination is a time-consuming process (1), and translation pausing sites at the ends of leader peptides ensure sufficient detention of ribosomes for repression of competing secondary structures (17). Concomitantly, all terminators of these attenuators are relatively distal (>20 bp) to the leader peptide gene, which ensure that ribosome pausing at the end of the leader peptide does not directly repress Rho-independent terminator.

### Function of sequences that precede terminators

Although the tendencies of TE–distance relations of W- and R-series were similar, precise TEs of the two series of plasmids differed for the same distances of <30 bp (Figure 1B). Moreover, RNAP dynamics are highly sequence specific (50), suggesting that sequence-specific TE–distance relations originate from varied RNAP dynamics on different insertion sequences, and hence, differing distance distributions between ribosomes and RNAP at termination sites. Thus, in addition to terminator sequences, both positions and sequences preceding terminators may be subject to evolutionary stress.

For partial terminators located in intergenic regions of the operon (24), precise measurements of TE are required for determination of expression ratio between upstream and downstream genes. In addition, readthrough of terminators produces antisense RNA (27), allowing the use of terminator efficiencies as a measure of antisense RNA expression. The present data indicate that 3′-end sequences of upstream genes also influence TEs by modulating the dynamics of ribosomes and RNAP. Thus, we suggest that both sequences at 3′-ends of upstream genes and sequences between genes and terminators are important experimental subjects for precise measurements of TE. Moreover, different TEs at different directions for bidirectional terminators may reflect differing distances to upstream genes or differing preceding sequences.

Cooperation between ribosomes and RNAP is a more important factor in determining TEs of Rho-independent terminators than previous studies indicate. Accordingly, fine tuning of TEs may be achieved by altering sequences or lengths of sequences that precede terminators, comprising a regulatory mechanism for protein ratios (24), intensities of transcriptional interferences and antisense RNA expression (27). Fine-tuning of transcription flux may be required in synthetic biology. Stochastic coupling between transcription and translation gives a gradual increase of TE–distance relation. This feature offers a convenient way to fine tune TE in synthetic biological studies. In order to perform this tuning, the insertion sequence should be designed with low GC-content and less consecutive GC to avoid secondary structure inhibiting terminator hairpin formation.

Different kinds of factors can affect TE of Rho-independent terminator, including transcription strength of upstream gene (56), regulations of different transcriptional factors (58), terminator position, upstream and downstream (7) context sequences, transcriptional coupled translation dynamics, U-tract composition (52), terminator hairpin stability and hairpin folding efficiency. Influences of many of these factors are still not clear and many of them may interfere with each other. Predicting TE of Rho-independent terminator precisely is still a big challenge and need more detail information further.

## SUPPLEMENTARY DATA

Supplementary Data are available at NAR Online.

SUPPLEMENTARY DATA
