# Supplementary material for: Effects of cooperation between translating ribosome and RNA polymerase on termination efficiency of the Rho-independent terminator
Source: Nucleic Acids Res. 2015 Nov 23;44(6):2554–63. doi: 10.1093/nar/gkv1285 (PMC4824070; doi:10.1093/nar/gkv1285)
Supplement: SUPPLEMENTARY DATA [file supp_44_6_2554__index.html]

Effects of cooperation between translating ribosome and RNA polymerase on termination efficiency of the Rho-independent terminator — Effects of cooperation between translating ribosome and RNA polymerase on termination efficiency of the Rho-independent terminator — SUPPLEMENTARY DATA 

# Effects of cooperation between translating ribosome and RNA polymerase on termination efficiency of the Rho-independent terminator

## SUPPLEMENTARY DATA

- SUPPLEMENTARY DATA
