# Supplementary material for: Effects of cooperation between translating ribosome and RNA polymerase on termination efficiency of the Rho-independent terminator
Source: Nucleic Acids Res. 2015 Nov 23;44(6):2554–63. doi: 10.1093/nar/gkv1285 (PMC4824070; doi:10.1093/nar/gkv1285)
Supplement: SUPPLEMENTARY DATA [file supp_gkv1285_nar-02532-r-2015-File006.docx]

**Supplementary data**

**Effects of cooperation between translating ribosome and RNA polymerase on termination efficiency of the Rho-independent terminator**

Rui Li, Qing Zhang, Junbai Li and Hualin Shi. 2015

Correspondence to: shihl@itp.ac.cn

**Supplementary Materials and Methods**

**Construction of Plasmids.** We constructed reporter system that terminator eﬃciency (TE) can be got by measuring ﬂuorescences of RFP (*mCherry*) and GFP (*GFPmut2*). All plasmids were transformed in cell BW-RI (1) derived from *E. coli* K-12 BW25113 with a cassette *spr-lacI-TetR* inserted at the attB site of chromosome to provide constitutive expression *lacI* and *TetR*.

All the plasmids (listed in table S1) were derived from plasmid pZE12-mcherry with colE1 replication ori and was marked by ampicillin resistance (2). We inserted GFP downstream of mCherry and then inserted a 79 bp insulator sequence upstream RBS of *GFP* to avoid changing secondary structure around RBS of GFP by varying upstream sequences, thus we got the reference plasmid I21 which contains the whole operon structure and no terminator between RFP and GFP. This operon is driven by synthetic P*_Llac-O1_* promoter which can be induced by isopropyl β-D-thiogalactoside (IPTG). Terminators were inserted between RFP and insulator sequence (Figure 1A). Experiment details are followed below.

Plasmid I21 was constructed as the reference plasmid, with TE = 0. We ampliﬁed DNA sequence with RFP (mcherry) gene using the primer pair “mchUPUP”–“mchTDW” (primers details are all listed in table S2) and the templet plasmid pZE12-mcherry. The product was digested with restriction enzymes XhoI and BamHI, and inserted into the same sites of plasmid pUA66 (3), thus we got plasmid pEVII-mprg. And with pEVII-mprg as the templet we ampliﬁed DNA segment with the whole operon using the primer pair “UPMch”–“mgTDW”. After digested with enzyme *Xho*I and BamHI, we inserted it into the same sites in plasmid pZE12-mcherry, yielding plasmid pZE12-mprg. The fragment with insulator was got using PCR with the primer pair “ninsert2”–“ninsert2-r” which were also templates in PCR, and then was inserted between BamHI and SalI sites of plasmid pZE12-mprg, thus we got plasmid I21.

Plasmids for measuring TE were derived from I21. Well studied bacteria phage *λ* terminator tR2 was chosen to illustrate the relation between TE and terminator position. We inserted diﬀerent lengths of spacer sequences between *RFP* and tR2 terminator using the short sequence or long sequence method (both of these two methods will be described below). To study the sequence-speciﬁc eﬀect, W-series and R-series plasmids were constructed by inserting natural sequence proceeding tR2 terminator in phage *λ* genome or a random sequence between RFP and tR2 terminator (spacer sequences are listed in Table S3). The random sequence for R-series was chosen that cannot fold into secondary structures interfering tR2 terminator hairpin folding. The short sequence and long sequence methods are as follows. (a) Short sequence method: we inserted short length sequences with terminator and the spacer sequence in the sites of HindIII and BamHI of I21. The insertion sequences are from annealing two complementary oligonucleotides directly. The oligonucleotide was designed with proper ends so that the annealing sequences with overhangs of enzyme sites can be directly inserted in the template. The annealing method was as described in Supporting Information of Hao et al. (4). Complementary oligoes at 100 µM were resuspended in “annealing buﬀer” (10mM Tris at pH=8.0, 50 mM NaCl, 1 mM EDTA). Solutions were then mixed with equal volumes in 500µL tube and placed in 94°C for 5 min, and then slowly cool down to room temperature. Then the product were directly used for ligation with the template from HindIII/BamHI digested plasmid I21. (b) Long sequence method: using this method we can change the spacer length gradually. Primer pairs “W#”–“mgTDW” were used in PCR (#: same as correspondent plasmids; for R-series using “R#”). “W#” contain mutations that lengthen or shorten spacer sequence, “mgTDW” locates downstream of 3′ UTR of *GFP* gene. PCR products were puriﬁed and digested with HindIII and then AvrII, and the ﬁnal products were inserted in plasmid I21 in the same sites. In plasmid W48, we introduced the translation initiation site of NinA gene of phage *λ.* We used long sequence to mutate RBS or start codon of this translation initiation site in plasmids W48-up1 and W48-up2, and the primers we used were “W48-up1”–“mgTDW” and “W48-up2”–“mgTDW”.

To check if translation is a key factor for repressing terminator we constructed plasmids W28p and W38p. Plasmids W28p and W38p were got by inserting a “G” upstream “TAA” stop codon of *RFP* gene in plasmid W28 and W38 respectively, and the new stop codon was ‘pushed’ downstream to 5 bp upstream of terminator hairpin for W28p and 38 bp downstream of terminator U-tract for W38p respectively. This mutation was introduced by primer “pushT” paired with “mChTUPUP” in PCR. After digested by HindIII and EcoRI, products were inserted in templates W28 and W38 in the same sites.

To study if changed translation strength can inﬂuence TE–distance relations, we change the original RBS into a new T710RBS7 from Lim et al. (5). Plasmids with this new RBS are marked with a suﬃx “r4”. We using the primer pair “r4-PCR”–“ttermdw” for PCR, then digested sequence was inserted with EcoRI/HindIII sites. And RBS T710RBS7 is introduced by primer “r4-PCR”. Thus we got plasmids I21-r4, W13-r4, W38-r4, R17-r4.

*RFP* gene and its RBS sequence are located between EcoRI and HindIII sites. To study the mechanism of repression of terminator, we delete part of *RFP* gene sequence and its RBS sequence only keeps the last 30bp of RFP using restriction enzyme EcoRI and HindIII sites as follows. The original sequence was replaced by a new insertion sequence with the last 30bp of RFP gene. Insertion sequence was annealed from two complementary oligonucleotides “30u-f” and “30u-r”. In this way, we got plasmids W28-30U and others with a sufﬁx “-30U”. And for plasmid W28p-30U we use oligonucleotide pair “30up-f” and “30up-r”. Same as W- and R-series, we also inserted 24 or 34 bp sequence between tR2 terminator and stop codon of modiﬁed RFP gene in plasmid W28p, insertion sequence for annealing of complementary oligonucleotide pairs “W28p-23-f”–“W28p-23-r” and “W28p-34-f”–“W28p-34-r”.

**Media, Growth and Measurements.** BW-RI cells with the appropriate plasmid were grown in M9 media with 0.5% glucose and appropriate amount of antibiotics at 37℃ to midlog phase. The cultures were diluted (1:250) in fresh media and cultured overnight. In the next morning the cultured were diluted to OD=0.002 in fresh media with antibiotics, glucose and 10 mM IPTG at saturation concentration for P*_Llac-O1_* promoter in wells of costa 48-well plate shaking in the speed of 170 rpm.

We measured cell growth by OD­_600_ and protein expressions by ﬂuorescences using a Wallac Victor3 1420 multilabel counter (PerkinElmer Life Sciences) every 30 minutes when cells were in exponential growth. Each data was got by averaging three repeated measurements. And for the same plasmid we measured at least three times.

For data analysis, *RFP* and *GFP* expression were analyzed as Leveau *et al.* (6). We only used data that cells were in exponential growth, i.e.，OD_600_: 0*.*034*−*0.33. We ﬁrst got doubling rate of cell [*µ*, slope of linear ﬁt of *log*_2_(OD_600_) vs. time], yielding a doubling time of about 1.2 hour for all cells in our experiments. Then we got *f* as the slope of linear ﬁt of ﬂuorescence vs. OD_600_ (ﬂuorescence background already removed, in our system, we used a second order polynomial ﬁtting the RFP and *GFP* ﬂuorescence background for BW-RI cell at different OD_600_). We computed ﬂuorescence production rates per growing cell per hour *P* = *fµ*(1 + *µτ/ln*2) (6), *τ* is maturation half time, we took 30 min for both mCherry (7) and *GFPmut2* (8). Due to cell all have a doubling time *∼* 1*.*2*h*, so *P ∝ f* .

**Quantitative Real-time PCR.** For quantitative Real-time PCR, stains were cultured in M9 media with 0.5% glucose and appropriate antibiotics to midlog phase. Then the cultures were diluted (1:250) in fresh media. After overnight growth, the cultures were diluted to *OD*_600_ = 0*.*002 in the identical fresh media with 10 mM IPTG in glass tubes, and we collected 5ml samples when cells growed to *OD*_600_*∼* 0*.*3. Doubling times of cells in glass tubes are almost the same as in 96-cell plate, about 1.2 h. Total RNA were extracted using TransGen EasyPure RNA Kit (TransGene; catalog no. ER101), and the residual DNA was removed by using Ambion Turbo DNA-free DNase (Ambio; catalog no. 1907). 1*µ*g total RNA was used for cDNA synthesis with PrimeScript II 1st Strand cDNA Synthesis Kit (TaKaRa; catalog no. D6210A). cDNA from 12 ng RNA (for RFP and *GFP*) was used for quantiﬁcation in 25µl reaction with Roche FastStart Universal SYBR Green Master (Roche; catalog no. 04913914001). rrsB, encoding 16S RNA, was as internal control; due to extreme abundance of 16SRNA, cDNA from 0.12 ng total RNA was used in 25 µl reaction. And real-time PCR was performed in eppendorf mastercycler ep gradient realplex4 real-time PCR system.

For data analysis, mRNA levels of *RFP* and *GFP* for strain with plasmid I21 were set to 1, for other plasmids mRNA levels were shown relative with mRNA levels of I21. Eﬃciency of replication for each amplicon was determined by series dilution method. And ratio of targets to rrsB were computed as the relative mounts of mRNA expression levels using corrected eﬃciency.

**Reference**

- 1. Levine E., Zhang Z., Kuhlman T., Hwa T. (2007) Quantitative Characteristics of Gene Regulation by Small RNA. *PLoS Biol.* 5:e229.
  2. Lutz R., Bujard H. (1997) Independent and tight regulation of transcriptional units in Escherichia coli via the LacR/O, the TetR/O and AraC/I_1_-I_2_ regulatory elements. *Nucleic Acids Res.* 25:1203–1210.
  3. Zaslaver A. *et al.* (2006) A comprehensive library of fluorescent transcriptional reporters for Escherichia coli. *Nat. Methods* 3:623–628.
  4. Hao Y. *et al.* (2011) Quantifying the sequence-function relation in gene silencing by bacterial small RNAs. *Proc. Natl Acad. Sci. USA* 108:12473–12478.
  5. Lim H.N., Lee Y., Hussein R. (2011) Fundamental relationship between operon organization and gene expression. *Proc. Natl Acad. Sci. USA* 108:10626–10631.
  6. Leveau J.H.J, Lindow S.E. (2001) Predictive and Interpretive Simulation of Green Fluorescent Protein Expression in Reporter Bacteria. *J Bacteriol.* 183:6752–6762.
  7. Cormack B.P., Valdivia R.H., Falkow S (1996) FACS-optimized mutants of the green fluorescent protein (GFP). *Gene* 173:33–38.
  8. Merzlyak E.M. *et al.* (2007) Bright monomeric red fluorescent protein with an extended fluorescence lifetime. *Nat. Methods* 4:555–557.

**Figure S1.** (A) Secondary structure of tR2 terminator, the first 4 nt sequence is chosen from W series. (B) RFP expression from plasmids under conditions of IPTG saturation. Experimental data are listed in Table S4.

.

**Figure S2.** Half the RBS strength don’t change TE. Plasmids with altered RBS are shown in red column, original ones are shown in black. (A) RFP expression level of plasmids and correspondent r4 series with a changed RBS r4, the expression level is normalized to the level of I21. (B) TE didn’t change after changing RBS. I21-r4 is as reference for calculation TE for r4 plasmids. Numerical data are listed in Table S4 and S5.

# Table S1. Bacteria plasmids used in this study

| Plasmid | Genotype | | Derived from | | Comments |  |
| --- | --- | --- | --- | --- | --- | --- |
| pUA66 | *PLlacO−*1: *GFPmut2* |  | | Kan marker, details see ref.(1) | | |
| pZE12-mcherry | *PLlacO−*1:*mCherry* | pZE12 (2) | | colE1 *ori,* Amp marker | | |
| pEVII-mprg | *PLlacO−*1:*mCherry*,*GFPmut2* | pUA66 | | Kan marker | | |
| pZE12-mprg | *PLlacO−*1:*mCherry*,*GFPmut2* | pZE12-*mCherry* | | colE1 *ori*, Amp marker | | |
| pZE12-I21 | *PLlacO−*1:*mCherry*,*GFPmut2* | pZE12-mprg | | colE1 *ori*, Amp marker, inserted a 79 bp random sequence upstream RBS of *GFP* in plasmid pZE12-mprg | | |
|  |  |  | |  |  |  |
| pZE12-tR2-W# | #:8,13,18,23,28,38,43,48,53 | pZE12-I21 | | W-series plasmids, tR2 terminator located between *RFP* and *GFP* | | |
|  |  |  | |  |  |  |
| pZE12-tR2-R# | #:9,17,24,32,39,44,49,54,59 | pZE12-I21 | | R-series plasmids, tR2 terminator located between *RFP* and *GFP* | | |
|  |  |  | |  |  |  |
| pZE12-tr2-W48-up1 | RBS of *NinA* silenced | pZE12-tR2-40 | | translation initiation of NinA is eliminated | | |
|  |  |  | |  |  |  |
| pZE12-tr2-W48-up1 | ‘AUG’ start codon of *NinA* mutated | pZE12-tR2-40 | | translation initiation of NinA is eliminated | | |
|  |  |  | |  |  |  |
| pZE12-tR2-28p | there a ‘G’ before ‘TAA’ stop codon of  *RFP* | pZE12-tR2-W28 | | modiﬁed *RFP* with a tag, stop codon 5 bp upstream tR2 terminator  hairpin | | |
|  |  |  | |  |  |  |
|  |  |  | |  |  |  |
| pZE12-tR2-28p-# | #:24,34 | pZE12-tR2-W28p | | inserted 24 or 34 bp between tR2  terminator and modiﬁed *RFP* of  W28p | | |
|  |  |  | |  |  |  |
|  |  |  | |  |  |  |
| pZE12-tR2-38P | there a ‘G’ before ‘TAA’ stop codon of RFP | pZE12-tR2-W38 | | modiﬁed *RFP* with a tag, stop codon 38 nt downstream terminator  U-tract | | |
|  |  |  | |  |  |  |
|  |  |  | |  |  |  |
| pZE12-I21-r4 | RBS of *RFP* changed to T710RBS7 (3) | pZE12-I21 | | translation initiation is about 60% of original one | | |
|  |  |  | |  |  |  |
| pZE12-tR2-W13-r4 | RBS of *RFP* changed to T710RBS7 (3) | pZE12-tR2-W13 | | translation initiation is about 60% of original one | | |
|  |  |  | |  |  |  |
| pZE12-tR2-W38-r4 | RBS of *RFP* changed to T710RBS7 (3) | pZE12-tR2-W38 | | translation initiation is about 60% of original one | | |
|  |  |  | |  |  |  |
| pZE12-tR2-R17-r4 | RBS of *RFP* changed to T710RBS7 (3) | pZE12-tR3-R17 | | translation initiation is about 60% of original one | | |
|  |  |  | |  |  |  |
| pZE12-tR2-W28p-30U | RBS and coding region of *RFP* is change into the last 30 bp of *RFP* with one ‘G’  before original stop codon ‘TAA’ | pZE12-tR2-W28 | |  | | |
|  |  |  |  |  | | |
|  |  |  |  |  | | |
| pZE12-tR2-W28-30U | RBS and coding region of *RFP* is change into the last 30 bp of *RFP* | pZE12-tR2-W28 | |  | | |
|  |  |  | |  | | |
| pZE12-tR2-W8-30U | RBS and coding region of *RFP* is change into the last 30 bp of *RFP* | pZE12-tR2-W8 | |  | | |
|  |  |  | |  | | |
| pZE12-tR2-R17-30U | RBS and coding region of *RFP* is change into the last 30 bp of *RFP* | pZE12-tR2-R17 | |  | | |

1. Zaslaver A. *et al.* (2006) A comprehensive library of fluorescent transcriptional reporters for Escherichia coli. *Nat. Methods* 3:623-628.
2. Lutz R., Bujard H. (1997) Independent and tight regulation of transcriptional units in Escherichia coli via the LacR/O, the TetR/O and AraC/I_1_-I_2_ regulatory elements. *Nucleic Acids Res*. 25:1203-1210.
3. Lim H.N., Lee Y., Hussein R. (2011) Fundamental relationship between operon organization and gene expression. *Proc. Natl Acad. Sci. USA* 108:10626-10631.

# Table S2: Primers and oligonucleotides used in this study

| Primer | Sequence (5′ to 3′) | | Comments | |  |
| --- | --- | --- | --- | --- | --- |
| RTrrsB56-F | | tgcaagtcgaacggtaacag | | rrsB real-time PCR | |
| RTrrsB190-R | | ttgcgacgttatgcggtatt | | rrsB real-time PCR | |
| RT-MCH2-151-F  1-F | | ctgaaggtgaccaagggtg | | RFP real-time PCR | |
| RT-MCH2-249-R | | gcttcaagtagtcggggatg | | RFP real-time PCR | |
| RT*gfp*503-F | | gacacaacattgaagatggaagc | | GFP real-time PCR | |
| RT*gfp*601-R | | agggcagattgtgtggacag | | GFP real-time PCR | |
| ninsert2 | | Cgggatccgcaaggtcatgaaaccaagccgctaggtccggtaatgcggaatcgac | | both template and primer for amplifying insulator, with BamHI site | |
| ninsert2-r | | Aaagtcgaccgatctggttgtcactcactacattagtgtcggtcgattccgcattacc | | both template and primer for amplifying insulator, with SalI site | |
| mChTUPUP | | ccgaaaagtgccacctgacgtctaagaaacc | | Sense primer for sequencing and amplifying RFP | |
| mgTDW | | ctttcctgcgttatcccctgattctgtgg | | Anti-sense primer for sequencing and amplifying GFP | |
| tttermup | | gcctacaacgtcaacatcaagttggacatcac | | Sense primer for sequencing and amplifying RFP | |
| ttermdw | | gatctgggtatctcgcaaagcattgaagacc | | Anti-sense primer for sequencing amplifying RFP | |
| TR2-cut | | agcttaacaggcctgctggtaatcgcaggcctttttattg | | annealed for insertion, with overhang of HindIII (5′) and BamHI (3′) | |
| TR2-cutc | | gatccaataaaaaggcctgcgattaccagcaggcctgtta | | annealed for insertion, with overhang of BamHI (5′) and HindIII (3′) | |
| W13 | | ctgcccaagcttctaataacaggcctgctgg | | PCR primer to change spacer length, HindIII site underlined | |
| W23 | | ctgcccaagcttcattactgagctaataacaggcc | | PCR primer to change spacer length, HindIII site underlined | |
| W28 | | ctgcccaagcttatctacattactgagctaataacaggc | | PCR primer to change spacer length, HindIII site underlined | |
| W38 | | ctgcccaagcttaaagatgaccatctacattactgagc | | PCR primer to change spacer length, HindIII site underlined | |
| W43 | | ctgcccaagcttagttaaaagatgaccatctacattactg | | PCR primer to change spacer length, HindIII site underlined | |
| W48 | | ctgcccaagctttatggagttaaaagatgaccatc | | PCR primer to change spacer length, HindIII site underlined | |
| W53 | | cccaagcttcggtatatggagttaaaagatgaccatctac | | PCR primer to change spacer length, HindIII site underlined | |
| W48-up1 | | ctgcccaagctttatccagttaaaagatgaccatc | | Primer to introducing mutation mutates RBS of *NinA* | |
| W48-up2 | | ctgcccaagctttatggagttaaaagaagaccatc | | Primer to introducing mutation mutates start codon of *NinA* | |

*Continued on next page*

**Table S2** – *Continued from previous page*

| Primer | Sequence (5*′* to 3*′*) | | Comments | |  |
| --- | --- | --- | --- | --- | --- |
| R9 | | agcttctatcggcctgctggtaatcgcaggcctttttattg | | annealed for insertion, with overhang of HindIII (5′) and BamHI (3′) | |
| R9R | | gatccaataaaaaggcctgcgattaccagcaggccgataga | | annealed for insertion, with overhang of BamHI (5′) and HindIII (3′) | |
| R17 | | agcttaaactgaactatcggcctgctggtaatcgcaggcctttttattg | | annealed for insertion, with overhang of HindIII (5′) and BamHI (3′) | |
| R17R | | gatccaataaaaaggcctgcgattaccagcaggccgatagttcagttta | | annealed for insertion, with overhang of BamHI (5′) and HindIII (3′) | |
| R24 | | agcttgtcacagaaactgaactatcggcctgctggtaatcgcaggcctttttattg | | annealed for insertion, with overhang of HindIII (5′) and BamHI (3′) | |
| R24R | | gatccaataaaaaggcctgcgattaccagcaggccgatagttcagtttctgtgaca | | annealed for insertion, with overhang of BamHI (5′) and HindIII (3′) | |
| R32(II) | | cccaagcttcatatccagtcacagaaactgaactatcg | | PCR primer to change spacer length, HindIII site underlined | |
| R39(II) | | cccaagcttgtttactcatatccagtcacagaaactg | | PCR primer to change spacer length, HindIII site underlined | |
| R44 | | cccaagcttccaatgtttactcatatccagtcac | | PCR primer to change spacer length, HindIII site underlined | |
| R49 | | cccaagctttcggtccaatgtttactcatatccagtc | | PCR primer to change spacer length, HindIII site underlined | |
| R54 | | cccaagcttaatcgtcggtccaatgtttactcat | | PCR primer to change spacer length, HindIII site underlined | |
| R59 | | cccaagcttcttacaatcgtcggtccaatgtttac | | PCR primer to change spacer length, HindIII site underlined | |
| check2-25f | | agctttacattactgagctaataacacccgcacttaacccgcttcggcgggtttttgtttttg | | annealed for insertion, with overhang of HindIII (5′) and BamHI (3′) | |
| check2-25r | | gatccaaaaacaaaaacccgccgaagcgggttaagtgcgggtgttattagctcagtaatgtaa | | annealed for insertion, with overhang of BamHI (5′) and HindIII (3′) | |
| pushT | | cccaagcttaccttgtacagctcgtccatgc | | Used for PCR and adding a ‘G’ upstream ‘TAA’ stop codon of RFP, HindIII site underlined | |
| r4-PCR | | ccggaattctaactttaaggaggaaaaaaaatggtgagcaaggg | | Used for PCR and amplify RFP with RBS T710RBS7 (1), EcoRI site underlined | |
| 30u-f | | aattcaccggcggcatggacgagctgtacaagta | | annealed for insertion, with overhang of HindIII (5′) and BamHI (3′) | |
| 30u-r | | agcttacttgtacagctcgtccatgccgccggtg | | annealed for insertion, with overhang of BamHI (5′) and HindIII (3′) | |

*Continued on next page*

**Table S2** – *Continued from previous page*

| Primer | Sequence (5′ to 3′) | | Comments | |  |
| --- | --- | --- | --- | --- | --- |
| 30up-f | | aattcaccggcggcatggacgagctgtacaaggta | | annealed for insertion, with overhang of BamHI (5′) and HindIII (3′) | |
| 30up-r | | agcttaccttgtacagctcgtccatgccgccggtg | | annealed for insertion, with overhang of BamHI (5′) and HindIII (3′) | |
| 20P-23-f | | agcttatctacattactgagctaatcaagtctgtgcatatacataacaggcctgctggtaatcgcaggcctttttattg | | annealed for insertion, with overhang of BamHI (5′) and HindIII (3′) | |
| 20P-23-r | | gatccaataaaaaggcctgcgattaccagcaggcctgttatgtatatgcacagacttgattagctcagtaatgtagata | | annealed for insertion, with overhang of BamHI (5′) and HindIII (3′) | |
| 20P-34-f | | agcttatctacattactgagctaaagttctgaactcaagtctgtgcatatacataacaggcctgctggtaatcgcaggcctttttattg | | annealed for insertion, with overhang of BamHI (5′) and HindIII (3′) | |
| 20P-34-r | | gatccaataaaaaggcctgcgattaccagcaggcctgttatgtatatgcacagacttgagttcagaactttagctcagtaatgtagata | | annealed for insertion, with overhang of BamHI (5′) and HindIII (3′) | |

- 1. Lim H.N., Lee Y., Hussein R. (2011) Fundamental relationship between operon organization and gene expression. *Proc. Natl Acad. Sci. USA* 108:10626-10631.

# Table S3. Spacer sequences of W-series and R-series.

|  | **Length of spacer***∗* | **Spacer sequence between stop codon and terminator*†*** |
| --- | --- | --- |
| **Plasmid** |  |  |
| tR2-W8 | 8 | gcttAACA |
| tR2-W13 | 13 | gcttCTAATAACA |
| tR2-W18 | 18 | gcttCTGAGCTAATAACA |
| tR2-W23 | 23 | gcttCATTACTGAGCTAATAACA |
| tR2-W28 | 28 | gcttATCTACATTACTGAGCTAATAACA |
| tR2-W38 | 38 | gcttAAAGATGACCATCTACATTACTGAGCTAATAACA |
| tR2-W43 | 43 | gcttAGTTAAAAGATGACCATCTACATTACTGAGCTAATAACA |
| tR2-W48 | 48 | gcttTATGGAGTTAAAAGATGACCATCTACATTACTGAGCTAATAACA*‡* |
| tR2-W48-up1 | 48 | gcttTATCCAGTTAAAAGATGACCATCTACATTACTGAGCTAATAACA^a^ |
| tR2-W48-up2 | 48 | gcttTATGGAGTTAAAAGAAGACCATCTACATTACTGAGCTAATAACA^b^ |
| tR2-R9 | 9 | gcttCTATC |
| tR2-R17 | 17 | gcttAAACTGAACTATC |
| tR2-R24 | 24 | gcttGTCACAGAAACTGAACTATC |
| tR2-R32 | 32 | gcttCATATCCAGTCACAGAAACTGAACTATC |
| tR2-R39 | 39 | gcttGTTTACTCATATCCAGTCACAGAAACTGAACTATC |
| tR2-R44 | 44 | gcttCCAATGTTTACTCATATCCAGTCACAGAAACTGAACTATC |
| tR2-R49 | 49 | gcttTCGGTCCAATGTTTACTCATATCCAGTCACAGAAACTGAACTATC |
| tR2-R54 | 54 | gcttAATCGTCGGTCCAATGTTTACTCATATCCAGTCACAGAAACTGAACTATC |
| tR2-R59 | 59 | gcttCTTACAATCGTCGGTCCAATGTTTACTCATATCCAGTCACAGAAACTGAACTATC |
| tR2-W28p | 5 | taaca |
| tR2-W28p-23 | 23 | tcaagtctgtgcatatacataaca |
| tR2-W28p-34 | 34 | agttctgaactcaagtctgtgcatatacataaca |

*∗* Length of spacer is mentioned in main text as distance from stop codon of upstream ORF to terminator.

*†*Together with last 2 nt of upstream “TAA” stop codon of RFP, “gctt” in 5` every spacer sequence composes HindIII Restriction Enzyme site “AAGCTT”.

*‡*RBS and start codon of *NinA* protein are underlined.

^a^ RBS of NinA gene is mutated, mutated bases are underlined.

^b^ Start codon of NinA gene is mutated, mutated base is underlined.

# Table S4. Fluorescence measurements for terminators between RFP and GFP with upstream *RFP* expression levels normal.

**Fluorescence measurement∗ (RFU/OD/hr), [IPTG]=10mM**

**Plasmid *RFP* expression level *GFP* expression level Calculated Termination eﬃciency†**

| I21 | 9.73E+04 | ±1.65E+04 | 3.34E+05 | ±6.54E+04 | 0.000 | ±0.000 |
| --- | --- | --- | --- | --- | --- | --- |
| W8 | 1.24E+05 | ±5.33E+03 | 3.18E+05 | ±1.35E+04 | 0.246 | ±0.070 |
| W13 | 1.29E+05 | ±1.14E+04 | 2.27E+05 | ±1.61E+04 | 0.526 | ±0.032 |
| W18 | 1.28E+05 | ±5.52E+03 | 1.25E+05 | ±8.61E+03 | 0.734 | ±0.028 |
| W23 | 1.14E+05 | ±1.55E+04 | 7.52E+04 | ±1.23E+04 | 0.829 | ±0.015 |
| W28 | 1.32E+05 | ±9.86E+03 | 4.01E+04 | ±4.01E+03 | 0.916 | ±0.007 |
| W38 | 9.58E+04 | ±1.14E+04 | 7.79E+04 | ±2.71E+04 | 0.783 | ±0.056 |
| W43 | 9.43E+04 | ±3.49E+03 | 8.87E+04 | ±4.32E+03 | 0.742 | ±0.005 |
| W48-up1 | 1.01E+05 | ±8.92E+03 | 6.92E+04 | ±2.42E+04 | 0.862 | ±0.003 |
| W48-up2 | 1.23E+05 | ±4.74E+03 | 5.83E+04 | ±2.53E+03 | 0.870 | ±0.003 |
| R9 | 1.26E+05 | ±6.25E+03 | 3.71E+05 | ±1.04E+04 | 0.167 | ±0.023 |
| R17 | 1.32E+05 | ±1.11E+04 | 3.57E+05 | ±1.78E+04 | 0.235 | ±0.020 |
| R24 | 1.36E+05 | ±5.67E+03 | 1.64E+05 | ±1.10E+04 | 0.660 | ±0.013 |
| R32 | 1.38E+05 | ±1.04E+04 | 7.98E+04 | ±4.30E+03 | 0.837 | ±0.010 |
| R39 | 1.15E+05 | ±1.20E+04 | 4.01E+04 | ±3.63E+03 | 0.904 | ±0.005 |
| R44 | 1.16E+05 | ±3.07E+03 | 5.70E+04 | ±2.31E+03 | 0.859 | ±0.008 |
| R49 | 1.07E+05 | ±5.05E+03 | 5.49E+04 | ±2.55E+03 | 0.856 | ±0.007 |
| R54 | 1.02E+05 | ±8.31E+03 | 4.46E+04 | ±3.15E+03 | 0.878 | ±0.008 |
| R59 | 1.01E+05 | ±6.69E+03 | 4.07E+04 | ±2.37E+03 | 0.888 | ±0.004 |
| R44 | 1.16E+05 | ±3.07E+03 | 5.70E+04 | ±2.31E+03 | 0.859 | ±0.008 |
| R49 | 1.07E+05 | ±5.05E+03 | 5.49E+04 | ±2.55E+03 | 0.856 | ±0.007 |
| R54 | 1.02E+05 | ±8.31E+03 | 4.46E+04 | ±3.15E+03 | 0.878 | ±0.008 |
| R59 | 1.01E+05 | ±6.69E+03 | 4.07E+04 | ±2.37E+03 | 0.888 | ±0.004 |

* Error is displayed as ±standard deviation among three or more repeated measurements.

† Fluorescences of plasmid of I21 measured that day are as references.

# Table S5. Fluorescence measurement for plasmid with altered RBS sequence.

**Fluorescence measurement∗ (RFU/OD/hr), [IPTG] = 10mM**

**Plasmid *RFP* expression level *GFP* expression level Termination eﬃciency**†

| I21-r4 | 5.58E+04 | ±6.03E+03 | 3.17E+05 | ±3.93E+04 | 0.000 | ±0.000 |
| --- | --- | --- | --- | --- | --- | --- |
| W13-r4 | 7.28E+04 | ±1.48E+03 | 1.97E+05 | ±1.15E+04 | 0.522 | ±0.032 |
| W38-r4 | 4.90E+04 | ±2.90E+03 | 3.54E+04 | ±2.68E+03 | 0.872 | ±0.007 |
| R17-r4 | 5.32E+04 | ±8.38E+03 | 2.51E+05 | ±2.99E+04 | 0.163 | ±0.030 |

∗Error is displayed as ±standard deviation among three or more repeated measurements.

† Fluorescences of plasmid of I21-r4 are as references.

# Table S6. Fluorescence measurements for pushT plasmids and W48.

**Fluorescence measurement∗ (RFU/OD/hr), [IPTG] = 10mM**

**Plasmid *RFP* expression level *GFP* expression level *TE_GFP_***†

| W28p | 5.41E+04 | ±1.44E+04 | 2.85E+05 | ±3.33E+04 | 0.152 | ±0.070 |
| --- | --- | --- | --- | --- | --- | --- |
| W28p-23 | 6.38E+04 | ±8.51E+03 | 1.62E+05 | ±8.13E+03 | 0.500 | ±0.025 |
| W28p-34 | 6.78E+04 | ±2.78E+03 | 7.64E+04 | ±4.61E+03 | 0.764 | ±0.014 |
| W38p | 3.78E+03 | ±9.05E+02 | 1.95E+05 | ±1.49E+04 | 0.400 | ±0.046 |
| W48 | 4.82E+04 | ±6.70E+03 | 9.44E+04 | ±8.35E+03 | 0.721 | ±0.021 |

*∗*Error is displayed as ±standard deviation among three or more repeated measurements. Lowered *RFP* expressions level for plasmids with “p” are due to changed RFP fluorescence by modifications of *RFP* protein gene (details see the section construction of plasmids.).

*†GFP* ﬂuorescence of plasmid of I21 is as reference.

# Table S7. Fluorescence measurements for 30U plasmids.

|  | **Fluorescence measurement**∗  **(RFU/OD/hr), [IPTG]=10mM** | |  |  |
| --- | --- | --- | --- | --- |
|  |  |  |  |  |
| **Plasmid** | ***GFP* expression level** | | ***TE_GFP_***† | |
| I21-30U | 3.52E+05 | ±1.25E+04 | 0.000 ± | 0.000 |
| W28-30U | 7.97E+04 | ±1.63E+03 | 0.773 ± | 0.005 |
| W28p-30U | 6.08E+04 | ±1.92E+03 | 0.827 ± | 0.005 |

*∗*Error is displayed as ±standard deviation among three or more repeated measurements.

†*GFP* ﬂuorescence of plasmid of I21-30U is as reference.

# Table S8. Real-time PCR measurements of selected plasmids.

**mRNA abundance∗**

| **Plasmid** | ***RFP*** | ***GFP*** | **calculated TE**† |
| --- | --- | --- | --- |
| I21 | 1 | 1 | 0 |
| W13 | 0.9363 | 0.53332 | 0.4304 |
| W23 | 0.70775 | 0.06515 | 0.90794 |
| W28 | 0.79778 | 0.06387 | 0.91994 |
| W43 | 1.07015 | 0.11013 | 0.89709 |
| W48 | 0.78895 | 0.13536 | 0.82844 |
| W53 | 0.68096 | 0.14791 | 0.78279 |
| W28p | 1.15258 | 0.79278 | 0.31217 |
| W38-r4 | 0.97585 | 0.10077 | 0.89673 |

∗ mRNA abundance of *RFP* and *GFP* were measured by quantitative real-time PCR, with promoter fully induced with 10mM IPTG, and 16s RNA is as internal control. Details see SI materials and methods.

† RNA abundances of plasmid I21 are as references.

# Table S9. Terminator positions relative with upstream leader peptides gene in attenuators

| **Peptide** | **Length of lead peptide (bp)** | **Distance from stop codon to terminator (bp)** † |
| --- | --- | --- |
| *trpL* | 45 | 43 |
| *thrL* | 60 | 30 |
| *leuL* | 87 | 24 |
| *ivbL* | 99 | 27 |
| *pryBI* | 135 | -46 |

† Positive distance mean downstream of the stop codon, minus distance means upstream of the stop codon
